# Inhibition of hepatitis B virus (HBV) gene expression and replication by *HBx* gene silencing in a hydrodynamic injection mouse model with a new clone of HBV genotype B

**DOI:** 10.1186/1743-422X-10-214

**Published:** 2013-06-28

**Authors:** Lei Li, Hong Shen, Anyi Li, Zhenhua Zhang, Baoju Wang, Junzhong Wang, Xin Zheng, Jun Wu, Dongliang Yang, Mengji Lu, Jingjiao Song

**Affiliations:** 1 Department of Infectious Diseases, Union Hospital of Tongji Medical College, Huazhong University of Science and Technology, Wuhan, P.R. China; 2 Department of Infectious Disease, Anhui Provincial Hospital, No.9 Lujiang Road, Hefei, P.R. China; 3 Division of Clinical Immunology, Tongji Hospital, Tongji Medical College, Huazhong University of Science and Technology, Wuhan, P.R. China; 4 Animal Center, Tongji Hospital of Tongji Medical College, Huazhong University of Science and Technology, Wuhan, P.R. China; 5 Institute of Virology, University Hospital of Essen, University Duisburg-Essen, Essen, Germany

**Keywords:** Hydrodynamic injection, HBV mouse model, HBV genotype B, *HBx* gene silencing, Antiviral research

## Abstract

**Background:**

It has been suggested that different hepatitis B virus (HBV) genotypes may have distinct virological characteristics that correlate with clinical outcomes during antiviral therapy and the natural course of infection. Hydrodynamic injection (HI) of HBV in the mouse model is a useful tool for study of HBV replication *in vivo*. However, only HBV genotype A has been used for studies with HI.

**Methods:**

We constructed 3 replication-competent clones containing 1.1, 1.2 and 1.3 fold overlength of a HBV genotype B genome and tested them both *in vitro* and *in vivo*. Moreover, A HBV genotype B clone based on the pAAV-MCS vector was constructed with the 1.3 fold HBV genome, resulting in the plasmid pAAV-HBV1.3_B_ and tested by HI in C57BL/6 mice. Application of siRNA against *HBx* gene was tested in HBV genotype B HI mouse model.

**Results:**

The 1.3 fold HBV clone showed higher replication and gene expression than the 1.1 and 1.2 fold HBV clones. Compared with pAAV-HBV1.2 (genotype A), the mice HI with pAAV-HBV1.3_B_ showed higher HBsAg and HBeAg expression as well as HBV DNA replication level but a higher clearance rate. Application of two plasmids pSB-HBxi285 and pSR-HBxi285 expressing a small/short interfering RNA (siRNA) to the *HBx* gene in HBV genotype B HI mouse model, leading to an inhibition of HBV gene expression and replication. However, HBV gene expression may resume in some mice despite an initial delay, suggesting that transient suppression of HBV replication by siRNA may be insufficient to prevent viral spread, particularly if the gene silencing is not highly effective.

**Conclusions:**

Taken together, the HI mouse model with a HBV genotype B genome was successfully established and showed different characteristics *in vivo* compared with the genotype A genome. The effectiveness of gene silencing against *HBx* gene determines whether HBV replication may be sustainably inhibited by siRNA *in vivo*.

## Introduction

Hepatitis B virus (HBV) causes acute and chronic infection in the human liver and subsequently hepatic cirrhosis and hepatocellular carcinoma (HCC) that severely affects human health [1-3]. Although a highly effective vaccine is now available for the prevention of new HBV infections, about 400 million people worldwide have already been chronically infected and suffer from chronic liver injury [4].

An immunologically well-characterized small animal model for HBV infection remains unavailable due to the strict host specificity of HBV infection, which greatly hampers HBV-related research. The laboratory mouse is genetically and immunologically defined, and a large collection of genetically modified animals exists. However, mice can not be infected with HBV. Several lines of transgenic mice with replication competent HBV genomes have been established and represented to be powerful tools for HBV research [5]. However, HBV replication in the transgenic mice is generated from the integrated HBV sequence harbored in all hepatocytes, which is different from what occurs during a natural infection [5-8]. The presence of HBV genomes in these mouse lines inevitably induces immune tolerance to the HBV antigens. In addition, the capability of production of transgenic mouse model is not readily available in ordinary laboratory conditions. Transplant mouse models were established and used for different studies [9-11]. However, the models are based on immunodeficient mouse strains and difficult to handle in the laboratory.

Hydrodynamic injection (HI) of replication-competent HBV DNA into the tail veins of mice can establish HBV replication in the mouse liver [12,13]. In 40% of injected C56BL/6 mice, the persistence of HBV surface antigenemia (HBsAg) was greater than 6 months. HBV persistence is determined by the mouse genetic background and plasmid backbone [12]. The HBV HI mouse model is a highly interesting model for testing vaccination strategies and mechanisms of viral persistence [14-18]. This model may be used to the study replication competence of HBV constructs [15]. Previously, the established HBV HI mouse models were based on HBV genotype A replication-competent clones [12,13]. As HBV genotypes B and C are highly prevalent in Asia and may have distinct virological characteristics that correlate with clinical outcomes in the natural course of infection and antiviral therapy, it is warranted to establish HBV HI mouse models with characterized HBV strains.

Patients with chronic HBV infection are currently treated with interferon alpha (IFN-α) or nucleotide analogs such as entecavir and tenofovir. However, the current therapies have only a limited success rate and frequent viral recurrence after cessation of therapy, therefore, new antiviral strategies are required [19,20]. RNA interference has been developed as potential therapeutic approaches [21-24]. In previous studies, siRNAs targeting different regions of the HBV genome were used. The *HBx* gene encodes a small non-structural protein that has diverse functions and is required for HBV efficient replication [25,26]. The *HBx* mRNA contains the common sequence of the four transcripts of HBV [25,27,28], which makes it a potentially useful target for antiviral therapy.

In this study, we addressed the question whether a replication competent HBV genotype B clone could replicate transiently and persistently in HI mouse model. Plasmids pSB-HBxi285 (non-viral vector) and pSR-HBxi285 (retroviral vector) that express siRNA targeting the *HBx* gene were constructed. Using these constructs, we asked how the gene silencing by *HBx* siRNA of genotype B influences the course of HBV replication and persistence in the HI mouse model.

## Results

### Identification of different folds of HBV DNA expression plasmids

The 1.1, 1.2 and 1.3 fold over-length HBV genome DNA including nt 1658-3215-1986, nt 1360-3215-1986 and nt 1040-3215-1986 were cloned into the pBluesript II KS (+) vector separately. The construction procedure is shown in Additional file 1: Figure S1A and schematic representation of the 1.1, 1.2 and 1.3 fold HBV genome are shown in Additional file 1: Figure S1B. The plasmids were analyzed by restriction enzyme digestion with *Pst*I and *Sac*I. *Pst*I and *Sac*I restriction digestion of pBS-HBV1.1_B_, pBS-HBV1.2_B_ and pBS-HBV1.3_B_ resulted in the appearance of additional bands of 3.5, 3.8 and 4.2 kb in addition to the pBluescript II KS (+) vector, respectively (Additional file 2: Figure S2). Consistently, *Pst*I restriction digestion of pBS-HBV1.1_B,_ pBS-HBV1.2_B_ and pBS-HBV1.3_B_ confirmed that these vectors have the sizes of 6.5, 6.8 and 7.2 kb, respectively (Additional file 2: Figure S2). The HBV 1.3 fold over length genome DNA was amplified and sub-cloned into the *Not*I site of the pAAV-MCS vector (Agilent technologies, La Jolla, USA). The construction procedure of pAAV-HBV1.3_B_ is shown in Additional file 1: Figure S1C. The plasmid pAAV-HBV1.3_B_ was identified by *Not*I restriction digestion and an additional band of 4.16 kb (data not shown).

### pBS-HBV1.3_B_ shows high replication competence both *in vitro* and *in vivo*

**Figure 1:**
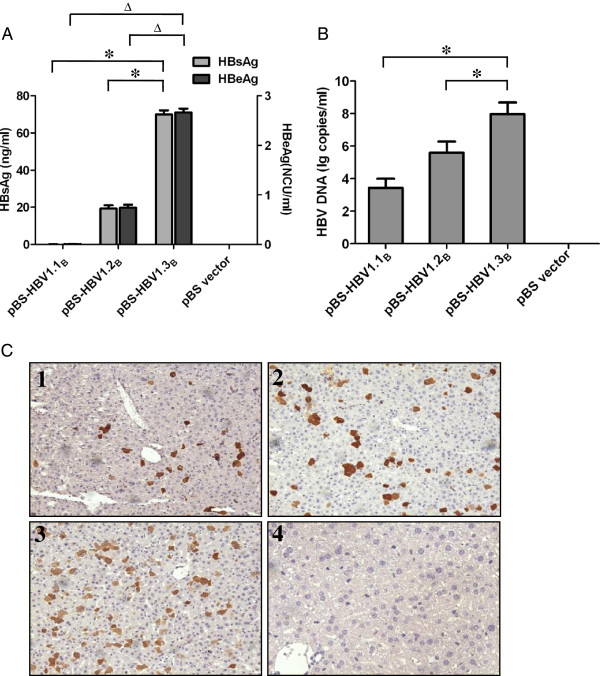
**Comparison of the *in vitro *and *in vivo *replication competence of pBS-HBV1. 1**_**B**_**, pBS-HBV1.2**_**B **_**and pBS-HBV1.3**_**B**_**.** 0.8 μg of plasmid DNA were transfected into Huh-7 cells which were seeded in 24-well plates at approximately 60% confluence and 10 μg of plasmid DNA were injected into the tail veins of BALB/c mice. Each group included 10 mice. **(A)** Titers of HBsAg (ng/ml) and HBeAg (NCU/ml, National Clinical Unit/ml,) in the supernatants of pBS-HBV1.1_B_, pBS-HBV1.2_B_ and pBS-HBV1.3_B_ transfected Huh-7 cells at 48 h post-transfection. The data were analyzed by one-way ANOVA, and the differences were statistically significant (* and ^Δ^ mean p < 0.01). **(B)** Real-time PCR detection of HBV DNA in mouse sera at day 7 after HI of pBS-HBV1.1_B_, pBS-HBV1.2_B_ and pBS-HBV1.3_B_ (the number of the mice ≥ 3). The data were analyzed by one-way ANOVA, and the differences were statistically significant (* means p < 0.01). **(C)** Immunohistochemical staining of the liver sections for HBcAg in hepatocytes of pBS-HBV1.1_B_- (1), pBS-HBV1.2_B_- (2), pBS-HBV1.3_B_- (3) and pBS (4)-injected mice at 7 day post injection (Original magnification: 200X).

To investigate the functionality of pBS-HBV1.1_B_, pBS-HBV1.2_B_ and pBS-HBV1.3_B_ in hepatoma cells, the plasmids were transfected into Huh-7 cells. At 48 h post-transfection, the culture supernatants of the transfected cells were harvested and subjected to HBsAg and HBeAg detection. As shown in Figure 1A, the HBsAg and HBeAg levels in the culture supernatant of pBS-HBV1.3_B_ transfected Huh-7 cells were significantly higher than those with the of pBS-HBV1.1_B_ and pBS-HBV1.2_B_ plasmids (p < 0.01).

To further compare the replication competence of pBS-HBV1.1_B_, pBS-HBV1.2_B_ and pBS-HBV1.3_B_*in vivo*, BALB/c mice were subjected to HI with these plasmids. Each group contained 10 mice. At 7 dpi, mouse serum samples were collected for HBV DNA quantification. The liver tissue was collected for immunohistochemical staining of HBcAg. Figure 1B showed that HI with pBS-HBV1.3_B_ resulted in a significantly higher HBV DNA level in mouse serum samples compared with HI with pBS-HBV1.1_B_ and pBS-HBV1.2_B_ (p < 0.01). Immunostaining of HBcAg demonstrated that HBcAg positive hepatocytes could be detected in the mouse liver after HI with each of the three plasmids but absent in the control mice. Moreover, the HBcAg positive hepatocytes were most abundant in the mouse liver of pBS-HBV1.3_B_ transfected mice (Figure 1C).

### The influence of the host genetic background and plasmid backbone on HBV persistence

It is reported that HBV persistence after HI in mice depends on the host genetic background and plasmid backbone [12].

**Figure 2:**
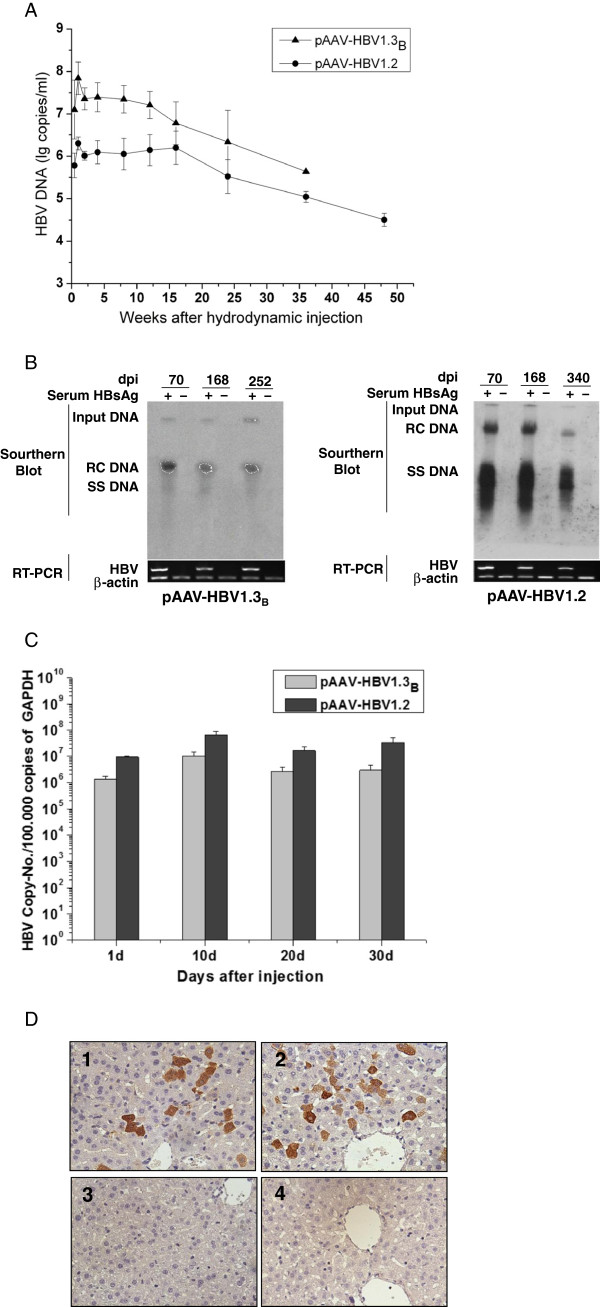
**HBV replication and gene expression *in vivo *after HI with pAAV-HBV1.3**_**B **_**or pAAV-HBV1.2.** 10 μg of pAAV-HBV1.3_B_ or pAAV-HBV1.2 were injected into the tail veins of C57BL/6 mice. pAAV-HBV1.3_B_ group included 14 mice and pAAV-HBV1.2 group included 9 mice. **(A)** Real-time detection of HBV DNA in mouse sera at the indicated time points (the number of the mice ≥ 3). **(B)** Southern blot analysis with 20 mg of total DNA extracted from liver tissues of the mice HI with pAAV-HBV1.3_B_ or pAAV-HBV1.2 at different time points after HI. All DNA samples were treated with RNase before subjected to agarose gel electrophoresis. Bands corresponding to the expected size of the input HBV plasmids, relaxed circular (RC) and single-stranded (SS) HBV DNAs are indicated. **(C)** Real-time PCR detection of HBV DNA levels in the liver of the mice after HI. **(D)** Immunohistochemical staining of the liver sections for HBcAg in hepatocytes of HBsAg-positive or HBsAg-negative mice at day 252 after HI with pAAV-HBV1.3_B_ (1, HBsAg positive mice and 3, HBsAg negative mice) and at day 340 after HI with pAAV-HBV1.2 (2, HBsAg positive mice and 4, HBsAg negative mice) (Original magnification: 400X).

Firstly, the 1.3 fold over-length HBV genome was cloned into pAAV vector to generate pAAV-HBV1.3_B_. HI with pAAV-HBV1.3_B_ was performed in C57BL/6 mice to examine whether this HBV isolate of genotype B is able to establish persistent replication *in vivo*. In parallel, pAAV-HBV1.2 described by Huang et al. (2006) was used as a reference. pAAV-HBV1.3_B_ group included 14 mice and pAAV-HBV1.2 group included 9 mice. The serum HBV DNA levels in C57BL/6 mice with pAAV-HBV1.3_B_ were determined at the indicated time points up to week 48 and were higher than those in mice with pAAV-HBV1.2 (Figure 2A).

The liver tissue was collected from C57BL/6 mice injected with pAAV-HBV1.3_B_ at 70, 168 and 252 dpi or pAAV-HBV1.2 at 70 dpi, 168 dpi and 340 dpi. HBV DNA was extracted from the liver tissue samples and assayed for HBV DNA by Southern blot analysis. Bands corresponding to the expected size of the input HBV plasmids and HBV replication intermediates including relaxed circular (RC) and single-stranded (SS) HBV DNAs could be detected in the liver of HBsAg-positive C57BL/6 mice hydrodynamically injected with pAAV-HBV1.3_B_ or pAAV-HBV1.2 (Figure 2B). HBV replication intermediates were at a higher level in mice injected with pAAV-HBV1.2 than in those with pAAV-HBV1.3_B._ Intrahepatic HBV DNA levels also detected by real-time PCR and the results were consistent with the Southern-blot results (Figure 2C). Immunohistochemical staining of the liver sections for HBcAg revealed that both cytoplasmic and nucleic HBcAg were detected in the liver of HBsAg-positive mice hydrodynamically injected with pAAV-HBV1.3_B_ or pAAV-HBV1.2 (Figure 2D). In contrast, no HBcAg positive cell was present in the liver of the control mice and HBsAg-negative mice. All results obtained in this experiment indicate that HBV genotype B construct was replication competent in the mouse liver.

**Figure 3:**
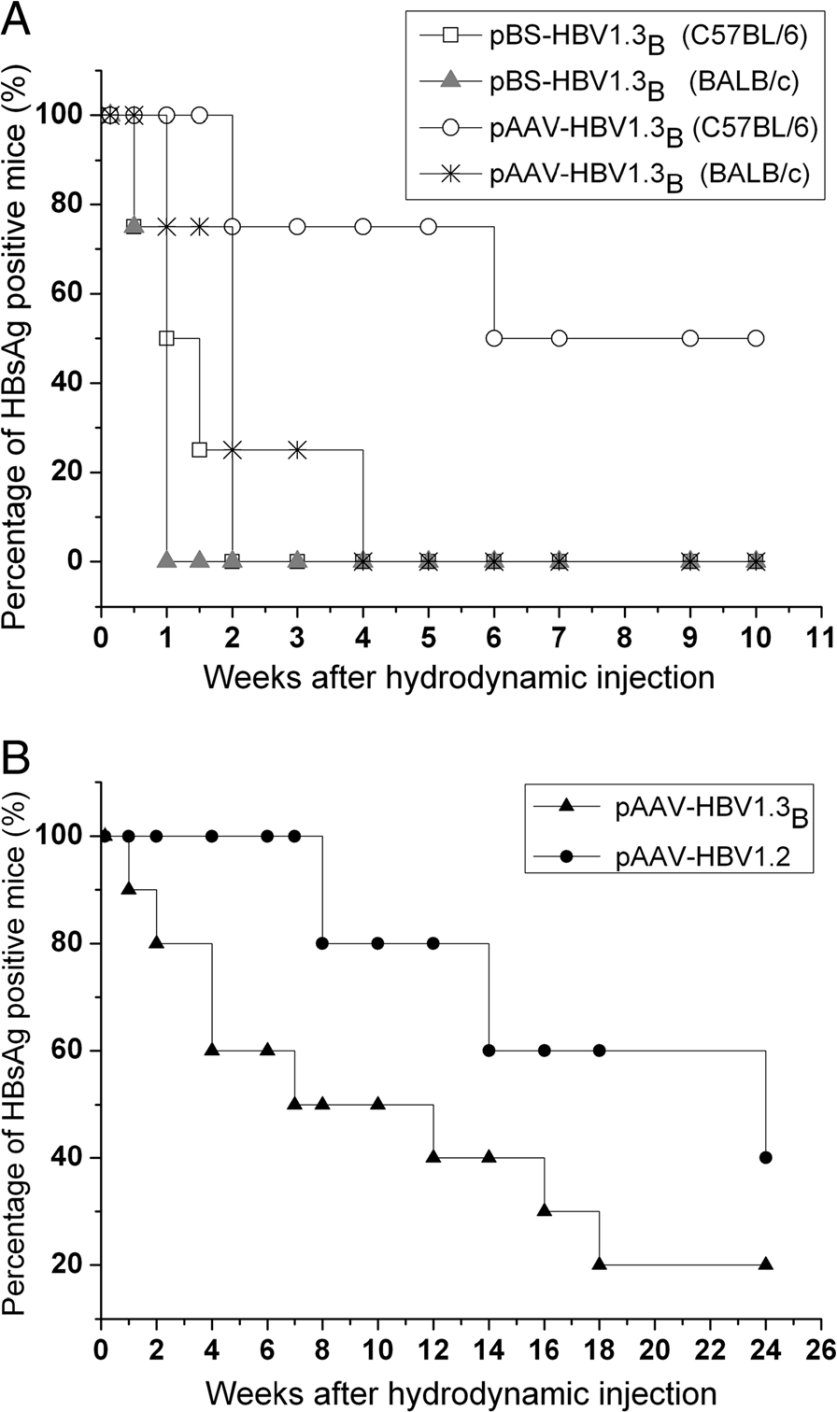
**HBV persistence after HI in mouse in dependence on the mouse genetic backgrounds and the vector backbones.** 10 μg of pBS-HBV1.3_B_ or pAAV-HBV1.3_B_ were injected into the tail veins of BALB/c or C57BL/6 mice. Each group included 5 mice. In addition, 10 μg pAAV-HBV1.3_B_ or pAAV-HBV1.2 were injected into the tail veins of C57BL/6 mice to test the ability of these two plasmids to establish persistent HBV replication. pAAV-HBV1.3_B_ group included 14 mice and pAAV-HBV1.2 group included 9 mice. **(A)** The positive rate for serum HBsAg in C57BL/6 or BALB/c mice receiving pBS-HBV1.3_B_ or pAAV-HBV1.3_B_ injections at indicated time points after injection. The data were analyzed by Kaplan–Meier analysis, and the differences between each group were statistically significant (p = 0.004, <0.05). **(B)** The positive rate for serum HBsAg in C57BL/6 mice receiving pAAV-HBV1.3_B_ or pAAV-HBV1.2 injection. The data were analyzed by Kaplan–Meier analysis, and the differences between two groups were statistically significant (p = 0.017, <0.05). The cutoff value for determining HBsAg-positivity was 0.1.

To investigate whether the vector backbone and the host genetic background also influence persistence of the HBV isolate of genotype B, 10 μg of the pBS-HBV1.3_B_ or pAAV-HBV1.3_B_ plasmids were injected hydrodynamically into the tail veins of male C57BL/6 or BALB/c mice. Each group included 5 mice. After HI, the mice were regularly bled and the temporal changes in HBsAg, HBeAg, HBcAb, HBsAb and HBV DNA levels were monitored. In BALB/c mice injected with pBS-HBV1.3_B_, the HBsAg level increased in the first three days but dropped quickly afterward and all mice became negative at 1 wpi. However, the HBsAg level in BALB/c mice injected with pAAV-HBV1.3_B_ declined slowly with time and 75% of the mice were HBsAg positive at day 10 dpi. HBsAg became undetectable in all mice at 4 wpi (Figure 3A). In contrast to BALB/c mice, the HBsAg level decreased much more slowly after injection of the same plasmids in C57BL/6 mice. 50% of C57BL/6 mice injected with pBS-HBV1.3_B_ were HBsAg-positive at 1 wpi and all mice became negative at 2 wpi. Moreover, 75% of C57BL/6 mice injected with pAAV-HBV1.3_B_ were still HBsAg-positive at 2 wpi. HBsAg remained detectable in 50% of the mice after 10 weeks (Figure 3A). These results confirmed that the host genetic background as well as the vector backbone influenced HBV persistence.

In addition, we also compared the kinetics of viremia between pAAV-HBV1.3_B_ and pAAV-HBV1.2 in the HI C56BL/6 mouse model. The serum HBV DNA level in pAAV-HBV1.3_B_ injected mice was higher than that in pAAV-HBV1.2 injected mice (Figure 2A). However, the C57BL/6 mice received pAAV-HBV1.3_B_ had a lower positive rate of HBsAg compared with that received pAAV-HBV1.2. 20% of the mice are HBsAg positive at week 24 after HI with pAAV-HBV1.3_B_, which was only half of that in mice injected with pAAV-HBV1.2 (Figure 3B).

None of the C57BL/6 mice injected with pAAV-HBV1.2 produced HBsAb after 28 dpi, although all of them produced HBcAb after the day 7 (Table 1). However, 3 of 14 C57BL/6 mice received pAAV-HBV1.3_B_ produced HBsAb after the 28 dpi. Moreover, the raise of HBsAb in pAAV-HBV1.3_B_ injected mice was faster than that in pAAV-HBV1.2. As shown in Table 1, for those mice producing HBsAb, they were HBsAg negative. All mice developed HBcAb after 7 dpi. Thus, different replication competent HBV genomes in the AAV vector may differ in their ability to establish the persistent replication in mice.

**Table 1:** **Appearance of anti**-**HBc and anti**-**HBs antibodies in C57BL**/**6 mice after HI of HBV plasmids**

	**Anti**-**HBc antibodies**		**Anti**-**HBs antibodies**		
**HBV clone**	**Day 1**	**day 7**	**Day 7**	**Day 28**	**Day 168**
**pAAV**-**HBV1**.**3**_**B**_	**0/14**	**14/14**	**0/14**	**3/14**	**6/8**
**pAAV**-**HBV1**.**2**	**0/9**	**9/9**	**0/9**	**0/9**	**4/8**

The number of C57BL/6 mice in each group positive for anti-HBc or anti-HBs antibodies after injected hydrodynamically with 10 μg of pAAV-HBV1.3_B_ or pAAV-HBV1.2 plasmids. All the mice with HBsAb are HBsAg negative.

### Impaired HBcAg specific cellular immunity in pAAV-HBV1.2 and pAAV-HBV1.3_B_ injected C57BL/6 mice during initial activation

**Figure 4:**
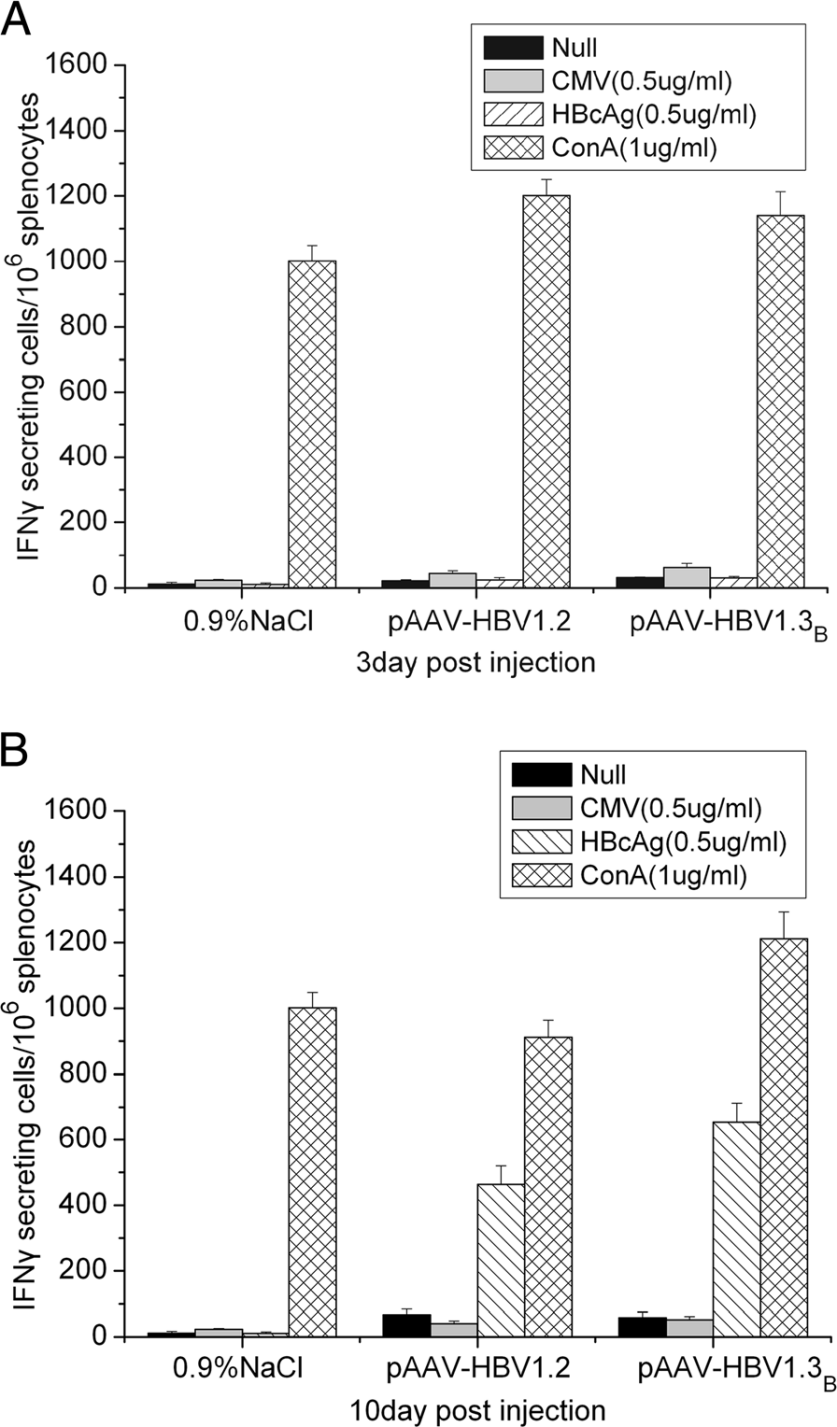
**Specific T-cell responses to HBcAg epitopes in mice after HI.** The specific T-cell responses to the full length HBcAg peptide after HI. The number of HBcAg specific IFNγ secreting cells in 1× 10^6^ splenocytes in pAAV-HBV1.2 and pAAV-HBV1.3_B_ injected mice at 3dpi **(A)** and 10dpi **(B)** by IFNγ ELISPOT assay in the presence of 0.5 μg/ml full length HBcAg peptide. The experiments were done in duplicate. The data were analyzed by *t* test, and there is no significant differences between those two groups (p > 0.05).

Huang et al. (2006) confirmed that the tolerance toward HBV surface antigen in this model was due to an insufficient cellular immunity against hepatitis B core antigen. To confirm this result, HBV-specific CTL responses against full length HBcAg peptide were detected at 3dpi and 10 dpi after HI by ELISPOT assay of IFN-γ producing cells. At 3 dpi we almost could not detect any significant levels of HBcAg specific IFNγ-producing cells in the splenocytes (Figure 4A). However, the average number of IFNγ secreting cells was 463 and 653 in 1× 10^6^ splenocytes in pAAV-HBV1.2 and pAAV-HBV1.3_B_ injected mice at 10dpi, respectively (Figure 4B). There is no significant difference between those two groups (p > 0.05). The result indicates that HBcAg-specific immune response may play a key role in clearing HBV infection at early stage in the HI mouse model.

### HBxi285 inhibited HBV replication *in vitro*

To apply RNAi *in vivo*, the vector based expression of siRNAs was explored in the HI mouse model. For these purpose, two different vectors pSuper.retro (pSR) and pSuper.basic (pSB) were tested. Two different target sequences in the HBV *x* gene were selected and cloned into the vectors (see Material and Methods).

**Figure 5:**
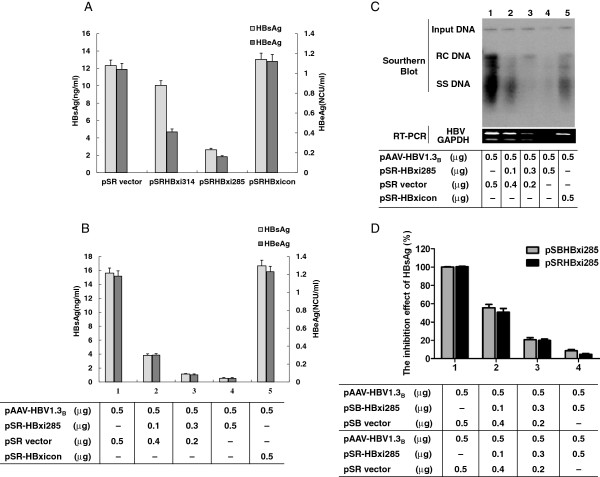
**The effect of *HBx *iRNA on HBV gene expression and replication *in vitro.*** Different concentrations of *HBx* iRNA and pAAV-HBV1.3_B_ were co-transfected into Huh-7 cells which were seeded in 24-well plates at approximately 60% confluence. The concentrations of the plasmids were indicated in the Tables. **(A)** HBsAg and HBeAg levels in the cell culture supernatants of cells co-transfected with 0.5 μg of pAAV-HBV1.3_B_- and 0.5 μg of pSR-HBxi314- or pSR-HBxi285. **(B)** HBsAg and HBeAg levels in the cell culture supernatants of cells co-transfected with pAAV-HBV1.3_B_ and 0, 0.1, 0.3, or 0.5 μg of pSR-HBxi285. **(C)** HBV replicative intermediates in Huh-7 cells transfected with pAAV-HBV1.3_B_ and 0, 0.1, 0.3, or 0.5 μg of pSR-HBxi285. **(D)** A comparison of the inhibition rate of HBsAg in the cell culture supernatants of cells co-transfected with pAAV-HBV1.3_B_- and pSB-HBxi285- or pSR-HBxi285.

The antiviral effect of pSR-HBxi314 and pSR-HBxi285 was investigated *in vitro*. As shown in Figure 5A, the HBsAg and HBeAg levels decreased by 80.2% and 78.7% when pAAV-HBV1.3_B_ and pSR-HBxi285 were co-transfected into Huh-7 cells, respectively. However, when pAAV-HBV1.3_B_ and pSR-HBxi314 were co-transfected into Huh-7 cells, the HBsAg and HBeAg levels were only inhibited by 10.5% and 30.3%, respectively. Different concentrations of pSR-HBxi285 were used to test whether the antiviral effect was dose-dependent. Figure 5B and C showed that pSR-HBxi285 inhibited HBsAg and HBeAg expression as well as HBV DNA in a concentration-dependent manner in the range of 0.1–0.5 μg. The 0.5 μg dose of pSR-HBxi285 was most effective for the reduction of HBsAg, HBeAg and HBV DNA in transient transfection.

To compare the antiviral effects of pSB-HBxi285 and pSR-HBxi285 *in vitro*, pAAV-HBV1.3_B_ and different concentrations of pSB-HBxi285 or pSR-HBxi285 were co-transfected into Huh-7 cells. Figure 5D shows that pSB-HBxi285 and pSR-HBxi285 inhibited HBsAg and HBeAg in a dose-dependent manner. However, there was no significant difference in their antiviral effect (P > 0.05).

### HBxi285 reduced serum HBsAg and HBV DNA levels in C57BL/6 mice after HI

**Figure 6:**
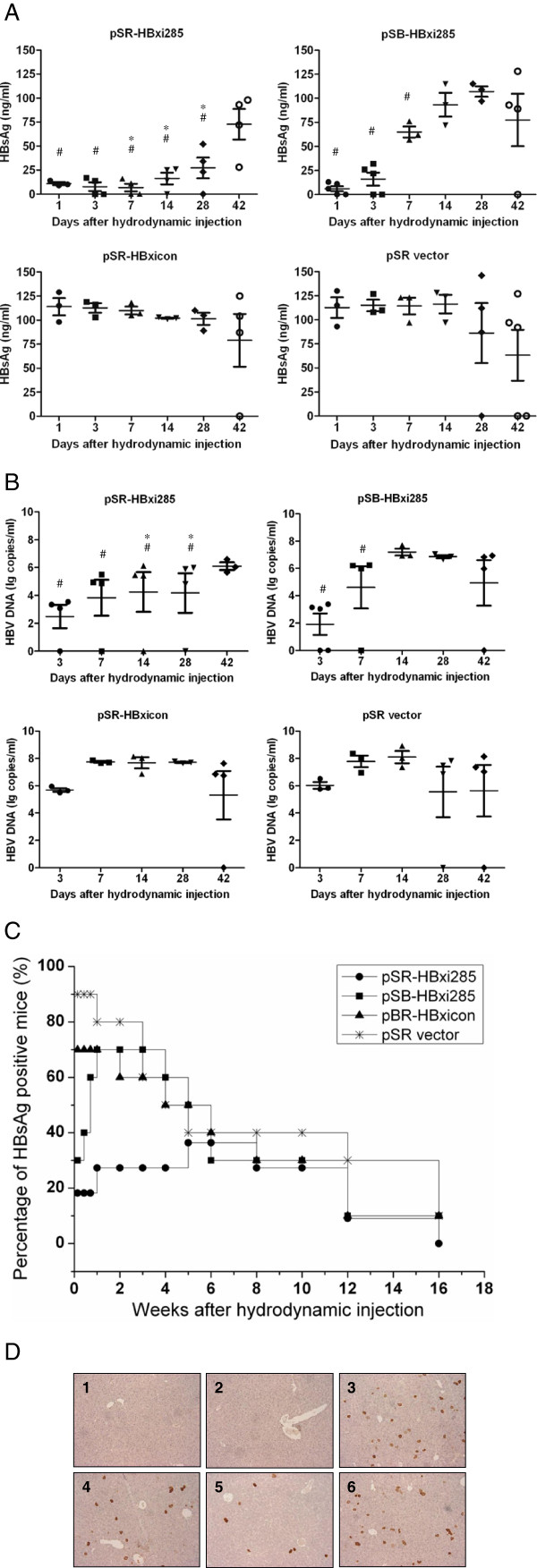
**The effect of HBxi285 on HBV gene expression and replication *in vivo. *** 10 μg pAAV-HBV1.3_B_ and 10 μg pSB-HBxi285, pSR-HBxi285 or control plasmids were co-injected into the tail veins of C57BL/6 mice. Each group included 10 mice. **(A)** HBsAg levels, **(B)** HBV DNA and **(C)** the positive rate of HBsAg in the serum of C57BL/6 mice treated with pAAV-HBV1.3_B_ and pSR-HBxi285 or pSB-HBxi285. Value 0 of HBV DNA means the copy number of HBV DNA is lower than detection limit. The data were analyzed by one-way ANOVA, and the differences were statistically significant (# : p < 0.05 pSR-HBxi285 group vs. pSR-HBxicon group; * : p < 0.05 pSR-HBxi285 group vs. pSB-HBxi285 group by one-way ANOVA) **(D)** Immunohistochemical staining for HBcAg in hepatocytes of C57BL/6 mice treated with pSR-HBxi285, pSB-HBxi285, pSR-HBxicon or pSR vector separately at 3 day post injection (1–4) and C57BL/6 mice treated with pSR-HBxi285 or pSR-HBxicon in the 4th wpi (5–6) (Original magnification: 200X).

Based on the previous experiments, 10 μg of pAAV-HBV1.3_B_ and 10 μg pSB-HBxi285 or pSR-HBxi285 were co-injected into C57BL/6 mice to determine the antiviral effect of HBxi285 *in vivo*. Each group contained 10 mice. Initially, HBsAg levels in mice were significantly reduced by HI in the presence of pSB-HBxi285 or pSR-HBxi285 plasmids at 1 dpi (p < 0.05), compared with the control groups receiving empty vectors (Figure 6A). However, the HBsAg levels in pSB-HBxi285 treated mice quickly increased during the follow up and reached the levels of the control at 14 dpi. In contrast, the HBsAg levels in pSR-HBxi285 treated mice were inhibited to a significantly lower degree before the 28 dpi compared with the control groups (p < 0.05). HBsAg became detectable in some mice at 42 dpi. It is worthy to note that the majority (7 of 10) of the mice received pSR-HBxi285 were HBsAg negative during the whole experimental period (Table 2). HBV DNA concentrations in mouse sera were determined and were well correlated with HBsAg levels (Figure 6B). Figure 6C shows that the HBsAg positive rate in pSB-HBxi285 or pSR-HBxi285 treated mice was significantly decreased in comparison to the controls at 1 dpi. Thereafter, the positive rate of HBsAg in pSB-HBxi285 treated mice increased gradually and showed no significant difference in comparison to the control at 7 dpi. The positive rate of HBsAg in pSR-HBxi285 treated mice remained at a lower level (18.2-27.3%) than other groups until the 4th week post injection (wpi) but showed similar levels to the other groups in the 5th wpi. Consistently, immunostaining of HBcAg in the liver section of mice after HI demonstrated that the co-application of pSR-HBxi285 or pBS-HBxi285 abolished HBcAg expression in the mouse liver at 3 dpi (Figure 6D). However, HBcAg may become detectable in the mouse liver which received pSR-HBxi285 similar to those of control mice 4 weeks after HI. The results indicated that HBxi285 could significantly inhibit the HBsAg production and HBV replication. However the vector pSR-HBxi285 was more efficient in reducing HBsAg and HBV DNA levels than that of pSB-HBxi285.

**Table 2:** HBsAg detection in HBxi285 treated and control mice

**Mouse group**	**Persistent negative**	**Negative** → **positive**^**a**^	**Persistent positive or positive** → **negative**^**b**^
**pSR**-**HBxi285**	**7/10**	**2/10**	**1/10**
**pSB**-**HBxi285**	**3/10**	**4/10**	**3/10**
**pSR**-**HBxicon**	**3/10**	**0/10**	**7/10**
**pSR vector**	**1/10**	**0/10**	**9/10**

^a^ Those mice with initial negative HBsAg became HBsAg positive. ^b^ Those mice with initial positive HBsAg became HBsAg negative during the whole experimental period of 16 weeks after HI. The cutoff value for determining HBsAg-positivity was 0.1.

## Discussion

In the present study, an HBV genotype B HI mouse model was established. After HI with pAAV-HBV1.3_B_, HBsAg and HBV DNA were detected in peripheral blood. Southern blot and immunohistochemical staining analysis revealed that HBV DNA and HBcAg were detected in the liver tissue of HBsAg-positive mice. Different hepatitis B virus genotypes may have distinct virologic characteristics, which may correlate with antiviral curative effects and clinical outcome [29,30]. The majority of HBV infections are distributed in the Asia-Pacific region. In these regions, patients are mainly infected with genotypes B and C. Previous studies showed that in China and Taiwan, approximately 90% of patients under thirty-five years old who are infected with HBV would develop HCC and most of them carried an HBV genotype B infection [30,31]. Now, we have a system at hand to examine the replication of HBV genotype B clone in the mouse models.

In present study, we investigated the effect of the HBV genome length on HBV replication competence to find the optimal structure of replication competent HBV genotype B clone. Three clones with 1.1, 1.2 and 1.3 fold over length HBV genotype B genomic DNA were constructed based on the pBluescript II KS (+) vector. Schematic representation of the 1.1, 1.2 and 1.3 fold HBV genome used in this study are shown in Additional file 1: Figure S1B. The plasmid with 1.3 fold genomes containing a complete extra copy of the entire EnhI, Enh II and *HBx* gene as shown in Additional file 1: Figure S1B which is a suitable HBV construct for transfection and HI experiments. When the cloned plasmids were transfected into Huh-7 cells or hydrodynamically injected into BALB/c mice, pBS-HBV1.3_B_ demonstrated significantly high-level replication and gene expression in comparison to pBS-HBV1.1_B_ and pBS-HBV1.2_B_ (Figure 1). Increasing the length of the 1.3 fold over length HBV genome did not further improve the replication competence. Nevertheless, the replication competence declined and could not reach the original level of HBV in nature infection state when the length is less than 1.2 fold of the HBV genome. Durantel et al. and other groups used 1.1 fold over length HBV genome for studies on drug resistant HBV isolates [32,33]. However, they added strong foreign promoters to enhance the HBV transcription and replication. In conclusion, the 1.3 fold HBV genome length containing a complete extra copy of the entire EnhI, EnhII and HBx gene as described in the present study could be regarded as an optimal length for HBV replication constructs with the authentic HBV sequence, independent on a foreign promoter.

To establish a persistent replication HBV mouse model with the HBV genotype B clone, 1.3 fold over length HBV genotype B genome was sub-cloned into the pAAV-MCS vector and introduced into C57BL/6 mice by HI. Huang et al. (2006) found that the AAV vector contributed to the persistence of HBV in mouse liver. Therefore, we used a similar vector pAAV-MCS, to construct HBV genotype B replicational clone in this study. This vector also has the ITR (inverted terminal repeats) structure, which was considered as a key factor for its stable existence *in vivo* by integration or non-integration way. Because the vector does not express other proteins except that encoded by the target genes, the vector backbone has no direct effect on the immune system.

After HI, the mice presented higher HBV DNA level, but a lower positive rate for HBsAg in serum compared with pAAV-HBV1.2 injected mice [12]. Apparently, HBV replication is not strictly correlated with HBsAg production, as known for patients [34-38]. The result indicated that the model is more robust when pAAV-HBV1.2 genotype A is used as compared with pAAV-HBV1.3_B_. Huang et al. (2006) confirmed that the tolerance toward HBV surface antigen in this model was due to an insufficient cellular immunity against hepatitis B core antigen, as documented in humans. We repeated some experiments and obtained the same results (Figure 4A and B). The difference in plasmid backbones between both clones needs to be investigated further.

The antigenemia and production of HBV particles were highly reproducible in the persistent HBV replication mouse model based on HI making this model appropriate for the evaluation of the efficacy of anti-viral drugs. Therefore, we further investigated the anti-viral effect of vector-expressed *HBx* siRNA in this model. Co-application of the *HBx* siRNA expression plasmids treatment of these mice induced a significant inhibition of HBV replication and expression. When pAAV-HBV1.3_B_ was co-injected into the tail veins of C57BL/6 mice with either pSB-HBxi285 or pSR-HBxi285, HBV expression and replication were efficiently inhibited. There are two methods commonly used to silence target genes. Chemical synthesized siRNAs have a good specificity and are commonly used on cells by transfection. However, such siRNAs, if not chemically modified, are instable and eliminated rapidly *in vitro and in vivo*. The production of siRNAs, particularly with specific chemical modifications, is very expensive. Another approach is to use siRNA transcription vectors. The disadvantages to use such as retroviral vectors are, for example, the lower transfection efficiency and slow action compared with chemical synthesized siRNA. However, the vector-based approach has some advantages regarding the stability and costs. It is important to prolong the expression of siRNAs to maintain the inhibition of HBV. Moreover, when the pSuper.Retro vector was transfected into a packaging cell line such as 293 T cell line, it could produce retroviral supernatants and obtain a higher rate of stable cell integration (The Manual of pSuper RNAi system, OligoEngine, Washington, USA) [39,40].

HI of naked plasmid DNA is a simple yet effective in vivo gene delivery method into hepatocytes. The early reports suggested that a large portion of hepatocytes could be reached with this technique [41-44]. Therefore, it could be a useful method to test the efficacy of RNAi based antiviral therapies in the mouse model. Certainly, there is still a challenge how to transfer the knowledge gained in this system into human situation.

In this study, non-viral (pSuper.Basic) and retroviral vectors (pSuper.Retro) were used to construct shRNA (small hairpin RNA) expression plasmids [45] targeting the *HBx* gene of HBV. The pSuper.Retro vector contains the H1 promoter which is a RNA polymerase III like promoter and can transcript shRNA steadily, and showed persistent inhibitory effect *in vitro*[46,47]. The pSuper.Retro vector includes 3′ and 5′ terminal LTR structure of murine stem cell virus. The pSuper.Retro vectors can be transfected into a packaging cell line to produce retroviral supernatants and obtain a higher rate of stable cell integration. In addition, this vector contains an incomplete packaging signal and could not produce recombinant retroviruses (The Manual of pSuper RNAi system, OligoEngine, Washington, USA). pSuper.Basic is a pUC origin vector (The Manual of pSuper RNAi system, OligoEngine, Washington, USA) and used to compare the stability with pSuper.Retro *in vivo*. The difference of both vectors in the gene silencing effects is likely due to their stability *in vivo*. HBx protein plays an important role in HBV replication and HBV induced HCC [26,48]. Therefore, *HBx* became a good target gene for gene silencing in recent years. Importantly, as all HBV RNA transcripts share the same sequence in the *HBx* due to its unique transcription mechanism, siRNAs targeting the *HBx* region actually knock down all HBV RNAs, as shown in the previous studies [28,49,50].

The result showed that the siRNA targeting the region nt 285–303 of the *HBx* coding region has a stronger antiviral effect than that targeting the region nt 314–332. It is known that siRNA-specific features such as low G/C content, a bias towards low internal stability at the sense strand 3′-terminus, lack of inverted repeats, and sense strand base preference, interaction with the cellular machinery called RISC and so on are likely to contribute to efficient RNAi process. The relative influence of these parameters is not fully understood and now under investigation by many groups [51-56]. When pAAV-HBV1.3_B_ and pSB-HBxi285 or pSR-HBxi285 was co-injected into the tail veins of C57BL/6 mice, HBV expression and replication could be significantly inhibited by the first week. Thereafter, the antiviral effect declined in the pSB-HBxi285 treated mice, whereas the antiviral effect of pSR-HBxi285 was maintained for at least 4 weeks. These results indicated that the retroviral vector based *HBx* siRNA expression plasmid could much more effectively inhibit HBV expression and replication than the non-viral vector based *HBx* siRNA expression plasmids. Among the mice which received pSR-HBxi285, HBV replication was completely suppressed in the majority of mice, as no HBsAg was detectable for the whole experimental period of 16 weeks. However, HBV gene expression and replication may resume in some mice despite an initial delay, indicating that transient suppression of HBV replication by *HBx* siRNA may also fail to clear HBV completely in some individuals. It is worth to mention that the change of serum HBsAg and HBV DNA in the mice after the application with *HBx* siRNAs occurred with different kinetics. The reason may be the different requirement of HBsAg translation and HBV DNA replication. It is well established that only a small fraction of HBsAg is required for virion production [57]. In the patients with chronic HBV infection, HBV DNA loads do not correlated with HBsAg [58]. Our results suggest that measures to prolong the antiviral effect of the siRNA should be investigated in future studies.

## Conclusions

In summary, our established HBV genotype B HI mouse model is reliable and could be applied for studies on genetic and antiviral research of chronic HBV infection. The retroviral vector based *HBx* siRNA expression plasmids could efficiently inhibit HBV expression and replication, suggesting that an siRNA-based treatment strategy may be an additional therapeutic option for future clinical use in patients with chronic HBV infection.

## Materials and methods

### Construction of replication competent HBV genotype B clones

The plasmid pAAV-HBV1.2 which contains an 1.2 fold over-length HBV genotype A genome (nt 1400-3182-1987) [12], was kindly provided by Prof. Pei-Jer Chen. The plasmid pUC-HBV containing a full length genotype B HBV genome (1820-3215-1824 nt, GenBank accession number: AY220698.1) was kindly provided by Prof. Jiming Zhang (Fudan University, China). The construction procedure of pBS-HBV1.1_B_, pBS-HBV1.2_B_ and pBS-HBV1.3_B_ is shown in Additional file 1: Figure S1A. First, the large fragments of the genotype B HBV DNA nt 1658-3215-247, nt 1360-3215-247 and nt 1040-3215-247 were amplified by PCR with primers *hbv*1.1LF (tagged with *Pst*I site) and *hbv*LR (tagged with *Xba*I site), *hbv*1.2LF (tagged with *Pst*I site) and *hbv*LR, *hbv*1.3LF (tagged with *Pst*I site) and *hbv*LR, rsepectively. Those three fragments were inserted into the pBluescript II KS (+) (Invitrogen, Camarillo, USA) vector digested with *Pst*I and *Xba*I. The small fragment of HBV DNA nt 247–1986 was amplified using primers *hbv*SF (tagged with *Xba*I site) and *hbv*SR (tagged with *Sac*I site), and was inserted into the the pBluescript II KS (+) vector digested with *Xba*I and *Sac*I. The resulting plasmids including nt 1658-3215-1986, nt 1360-3215-1986 and nt 1040-3215-1986 of HBV genome were named as pBS-HBV1.1_B_, pBS-HBV1.2_B_ and pBS-HBV1.3_B,_ respectively. Schematic representation of the 1.1, 1.2 and 1.3 fold HBV genome are shown in Additional file 1: Figure S1B. All primers used to amplify HBV fragments are listed in Table 3. All DNA fragments in the final transfer vectors were amplified with Pyrobest DNA polymerase (TAKARA, Dalian, China) and cloned into the commonly used, commercially available pGEM-T vector (Promega, Madison, USA) for PCR cloning and sequencing. The correctness of the sequences of cloned DNA fragments were confirmed by sequencing. To establish HI mouse model with a high positive rate of HBsAg, we cloned the 1.3 fold HBV genome into the vector including the backbone of adeno-associated virus, similar to the described procedure [12]. The HBV 1.3 fold over length genome DNA was amplified from pGEM-HBV1.3_B_ with primers *hbv*1.3 F (tagged with *Not*I) and *hbv*1.3R (tagged with *Not*I), and sub-cloned into the *Not*I site of the pAAV-MCS vector (Agilent technologies, La Jolla, USA). The resulting plasmid was designated as pAAV-HBV1.3_B_. The construction procedure of pAAV-HBV1.3_B_ is shown in Additional file 1: Figure S1C.

**Table 3:** **Primers for amplification of 1**.**3**, **1**.**2**, **and 1**.**1 fold over length HBV replication**-**competent clones**

**Primer**	**Position**	**Sequence**	**Reference**^**a**^
*hbv*1.1LF	HBV 1658-1677	5′-aa*c tgc ag*a aga gga ctc ttg gac ttt c-3′	AY220698.1
*hbv*1.2LF	HBV 1360-1379	5′-aa*c tgc ag*a gta tac atc att tcc atg g-3′	AY220698.1
*hbv*1.3LF	HBV 1040-1058	5′-tgc aa*c tgc ag*t gga tat cct gct tta atg-3′	AY220698.1
*hbv*LR	HBV 253-233	5′-gc*t cta ga*c tct gtg gta ttg tga-3′	AY220698.1
*hbv*SF	HBV 247-264	5′-ggc tag *tct aga *ctc gtg gtg g-3′	AY220698.1
*hbv*SR	HBV 1986-1969	5′-acc *gag ctc*tcg aat aga agg aaa gaa-3′	AY220698.1
*hbv*1.3 F	pGEM-T 4-39	5′-ata aga at*g cgg ccg c*cg aat tgg gcc cga cgt cg-3′	X65308.2
*hbv*1.3R	pGEM-T 117-81	5′-ata gtt ta*g cgg ccg c*gt tgg gag ctc tcc cat atg-3′	X65308.2

Restriction sites are shown in italics. The gene sequences included in the primers are indicated by underling. ^a^ The Genbank accession numbers of the used reference sequences are given.

### Cell culture and transfection

Huh-7 cells (Human hepatocarcinoma cell line) were maintained in 1640 medium supplemented with 10% of fetal bovine serum, 100 μg/ml penicillin and 100 μg/ml streptomycin. Cells were seeded in 24-well plates at approximately 60% confluence. After 24 h, cells were transfected with Lipofectamine 2000 (Invitrogen, Camarillo, USA) according to the manufacturer’s instructions. For pBS-HBV1.1_B_, pBS-HBV1.2_B_ and pBS-HBV1.3_B_, 0.8 μg of plasmid DNA and 2 μl of lipofectamine 2000 were placed in each well of a 24-well plate. The efficiency of our transfection procedure was verified and controlled by transfection of a GFP expressing vector, as described previously [59].

To prove the antiviral effect of *HBx* siRNA, 0.5 μg of pAAV-HBV1.3_B_ and 0.5 μg of pSR-HBxi314 or pSR-HBxi285 were co-transfected into Huh-7 cells. The dose dependent antiviral effect of pSR-HBxi285 was tested by co-transfection of 0.5 μg pAAV-HBV1.3_B_ with 0, 0.1, 0.3 and 0.5 μg of pSR-HBxi285 into Huh-7 cells. To compare the antiviral effects of pSB-HBxi285 and pSR-HBxi285, 0.5 μg pAAV-HBV1.3_B_ combined with 0, 0.1, 0.3 and 0.5 μg of pSB-HBxi285 or pSR-HBxi285 were co-transfected into Huh-7 cells in parallel. All cell transfection experiments were performed three times.

### HI experiments

The replication competence of the overlength HBV genomes *in vivo* was tested by HI with pBS-HBV1.1_B_, pBS-HBV1.2_B_ or pBS-HBV1.3_B_ plasmids in BALB/c mice (female, 6–8 weeks old, from the breeding colonies of the experimental animal center in Hubei Province, China), with 10 mice per group. C57BL/6 mice (male, 6–8 weeks old, from the breeding colonies of the experimental animal center in Shanghai, China), were subjected to HI with pAAV-HBV1.3_B_ or pAAV-HBV1.2 to test the ability of these plasmids to establish persistent HBV replication. pAAV-HBV1.3_B_ group included 14 mice and pAAV-HBV1.2 group included 9 mice. To investigate the factors affecting HBV persistent in the mouse liver, we injected pBS-HBV1.3_B_ or pAAV-HBV1.3_B_ into BALB/c or C57BL/6 mice separately, with 5 mice per group. HI experiments were carried out as described previously [17], 10 μg of HBV plasmid DNA was injected into the tail veins of mice in a volume of 0.9% NaCl, equivalent to 8% of the mouse body weight. The total volume was delivered within 5–8 s. For *HBx* siRNA experiments, 10 μg pAAV-HBV1.3_B_ and 10 μg pSB-HBxi285, pSR-HBxi285 or control plasmids were co-injected into the C57BL/6 mice. Each group included 10 mice. The control mice were injected only with the volume of 0.9% NaCl, equivalent to 8% of the mouse body weight.

### Detection of HBsAg, HBeAg, HBsAb and HBcAb

Culture supernatants of pBS-HBV1.1_B_, pBS-HBV1.2_B_ or pBS-HBV1.3_B_ transfected Huh-7 cells were harvested at 48 h post-transfection. Mouse sera were collected at the 1d (day), 3d, 7d, 10d, 2w (week), 3w, 4w, 5w, 6w, 7w, 8w, 9w, 10w… after HI. The analysis of HBsAg (ng/ml), HBeAg (NCU/ml, National Clinical Unit/ml), HBsAb (mIU/ml, Milli-International Units) and HBcAb (S/Co, Sample/Cutoff value) levels were determined by using the Architect system and CMIA kits (Abbott Laboratories, Wiesbaden-Delkenheim, DE) according to the manufacturer’s instructions.

### Isolation and analysis of HBV DNA in mouse sera

HBV DNA was extracted from 40 μl of mouse serum samples. The protocol was described previously [17]: 40 μl of samples were treated with 10 μg DNase I for 16 h at 37°C. 100 μl of lysis buffer (20 mM Tris–HCl, 20 mM EDTA, 50 mM NaCl and 0.5%SDS) containing 50 μg proteinase K were added. After incubation at 65°C for 3 h, viral DNA was isolated by phenol/chloroform extraction and ethanol precipitation. The DNA pellet was rinsed with 70% ethanol and resuspended in 10 μl of ddH_2_O.

The quantification of HBV DNA was performed by using a routine real time PCR procedure, described previously [34,60-64]. The HBV copy numbers were determined by SYBR Green Real time PCR Master Mix (commercially available assay kit, TOYOBO, Osaka, Japan). Melt curve analysis and agarose gel electrophoresis were used to verify the specificity of the real-time PCR. The following primers were used: forward primer: 5′-CTG CAT CCT GCT GCT ATG-3′ (nt 408–425), reverse primer: 5′-CAC TGA ACA AAT GGC AC-3′ (nt 685–701) according to the reference sequence with Genbank accession number (AY220698.1). Serum containing a known concentration of HBV DNA was used as a positive control.

### Immunohistochemistry

The liver tissue was taken from the mice receiving HI with pBS-HBV1.1_B_, pBS-HBV1.2_B_ or pBS-HBV1.3_B_ at 7 day post injection (dpi) and the mice receiving HI with pAAV-HBV1.3_B_ at 70 dpi, 168 dpi and 252 dpi or pAAV-HBV1.2 at 70 dpi, 168 dpi and 340 dpi, which was then used for immunohistochemical staining of the HBcAg in hepatocytes. The liver tissue of the mice received 0.9% NaCl was used as negative control. Liver tissue was collected from the mice and embedded in paraffin. Intrahepatic HBcAg was visualized by immunohistochemical staining with rabbit anti-HBc (Dako) of the liver tissue sections. The liver tissue sections were also stained with hematoxylin.

### Purification and Southern blot analysis of HBV DNA from the liver tissue of mice

Total DNA from the pAAV-HBV1.3_B_ and *HBx* siRNA expression plasmids co-transfected Huh-7 cells and the liver tissue of mice was isolated using commercial kits (OMEGA, Norcross, USA). The isolated HBV DNA was subjected to agarose gel electrophoresis followed by denaturation and Southern blot analysis. All DNA samples were treated with RNase before gel electrophoresis. HBV DNA was detected by hybridization with a digoxigenin (DIG) (Roche, Lewes, UK)-labeled full length HBV probe. The hybridization signals were visualized on x-ray film using an alkaline phosphatase-anti-DIG-Fab conjugate in the presence of the chemiluminescence substrate CDP-Star (Roche).

### Enzyme-Linked Immunospot (ELISPOT) assay

ELISPOT assay was carried out using the mouse IFN-γ precoated ELISPOT Kit (Dakewe, Shenzhen, China) according to the manufacturer’s instructions. Briefly, 96-well flat-bottomed microtiter plates were preincubated with the coating antibody (anti-IFN-γ monoclonal antibody) at 4°C overnight and blocked for 2 hr at 37°C. Mouse splenocytes at the density of 1 × 10^6^ cells per well were added to wells in triplicate with final concentration of 0.5 μg/ml full length HBcAg peptide (ProSpec, Ness-Ziona, Israel) separately and incubated at 37°C, 5% CO_2_ for 24 hr. 1 μg/ml of ConA (Sigma, St. Louis, USA) was used as positive control. Thereafter, cells were removed. Wells were washed ten times with PBS containing 0.05% Tween-20 (PBS-T) and incubated with 100 ml of biotinylated anti-IFNγ antibody for 1 hr. The plates were washed again with PBS-T and incubated with 50 ml HRP-strepto-avidin solution at 37°C for 1 hr. Spot-forming cells were counted and analyzed with an ELISPOT plate reader (BioReader 4000, Biosys, Germany). Results were presented as spot-forming cells per 1 × 10^6^ cells.

### Construction of *HBx* siRNA expression plasmids

Two pairs of DNA sequences targeting the nt 285–303 and nt 314–332 of the *HBx* coding region of HBV (genotype B, adw) were designed in accordance with the protocols for the siRNA expression vectors pSuper.Basic and pSuper.Retro (OligoEngine, Washington, USA), and the two RNAi design server (http://www.ambion.com/techlib/misc/siRNA_tools.html and http://www.dharmacon.com/DesignCenter). The DNA sequences were as follows: HBxi314F, 5′-GAT CCC CCG AC*C GAC CTT GAG GCA TA*T TCA AGA GA*T ATG CCT CAA GGT CGG TCG* TTT TTA-3′, HBxi314R, 5′-AGC TTA AAA A*CG ACC GAC CTT GAG GCA TA*T CTC TTG AA*T ATG CCT CAA GGT CGG TCG* GGG-3′; HBxi285F, 5′-GAT CCC C *GA GGA CTC TTG GAC TTT CA*T TCA AGA GA*T GAA AGT CCA AGA GTC CTC* TTT T TA-3′, HBxi285R, 5′-AGC TTA AAA A*GA GGA CTC TTG GAC TTT CA*T CTC TTG AA*T GAA AGT CCA AGA GTC CTC* GGG-3′, the *HBx* iRNA control sequences were: HBxiconF, 5′-GAT CCC C*GC ACC TAT AAC AAC GGT AG*T TCA AGA GA*C TAC CGT TGT ATA GGT GC*T TTT TA-3′, HBxiconR, 5′-AGC TTA AAA A*GC ACC TAT AAC AAC GGT AG*T CTC TTG AA*C TAC CGT TGT TAT AGG TGC* GGG-3′ (the sequences targeting *HBx* gene are shown in italics). The single strand DNA products were purchased from Invitrogen (Shanghai, China).

The above complementary sequences were phosphorylated and ligated into *Bgl*II and *Hin*dIII digested pSuper.Basic or pSuper.Retro vector. The resulting plasmids were named as pSB-HBxi314, pSB-HBxi285, pSB-HBxicon, pSR-HBxi314, pSR-HBxi285 and pSR-HBxicon. The clones were sequenced to confirm the correctness of the sequences.

### Statistical analysis

The data were evaluated by Student’s t-test or one-way ANOVA followed by Tukey’s post hoc test. The statistical significance of the data was considered at p < 0.05.

### Approval of the ethics committee

The animal experiments were carried out in concordance with the guidelines established by the Institutional Animal Care and Use Committee at the Huazhong University of Science and Technique and the Tongji Hospital of Tongji Medical College. The mice used in this study were anesthetized with ketamine and xylazine.

## Competing interests

The authors declare that they have no competing interests.

## Authors’ contributions

LL, JS, DY, and ML conceived and designed the experiments. LL, JS, HS, and ZZ performed the experiments. LL, JS, DY, and ML analyzed the data. AL, BW, XZ, JW, JJW, DY, and ML contributed reagents/materials/analysis tools. JS, DY, and ML wrote the paper. All authors read and approved the final manuscript.

## Supplementary Material

Additional file 1: Figure S1 Construction procedure for the pBS-HBV1.1_B_, pBS-HBV1.2_B_, pBS-HBV1.3_B_ and pAAV-HBV1.3_B_ vectors. (**A**) The fragments containing 1.1, 1.2 and 1.3 fold over length HBV genomes were amplified from the plasmid pUC-HBV containing a full length genotype B HBV genome (1820-3215-1824 nt, GenBank accession number AY220698.1) and inserted into the *Pst*I and *Sac*I sites of the plasmid pBluescript II KS (+). (**B**) Schematic representation of the 1.1, 1.2 and 1.3 fold HBV genome used in this study are shown. HBV ORFs as well as enhancers are indicated in different patters. (**C**) The HBV 1.3 fold over length genome DNA was amplified from pGEM-HBV1.3_B_ and sub-cloned into the *Not*I site of the pAAV-MCS vector. Click here for file

Additional file 2: Figure S2 Identification of pBS-HBV1.1_B_, pBS-HBV1.2_B_ and pBS-HBV1.3_B_. pBS-HBV1.1_B_, pBS-HBV1.2_B_ and pBS-HBV1.3_B_ were identified by restriction enzyme analysis. Lines 1/9: 1 kb DNA ladder; Lines 2–4: pBS-HBV1.1_B_, pBS-HBV1.2_B_ and pBS-HBV1.3_B_ were digested with *Pst*I and *Sac*I respectively; Lines 5: pBluescriptII KS (+) was digested with *Pst*I and *Sac*I; Lines 6–8: pBS-HBV1.1_B_, pBS-HBV1.2_B_ and pBS-HBV1.3_B_ were digested with *Pst*I. Click here for file

## References

[B1] Lai CL Ratziu V Yuen MF Poynard T Viral hepatitis B Lancet 2003 10 2089 2094 10.1016/S0140-6736(03)15108-2 14697813

[B2] Ganem D Prince AM Hepatitis B virus infection—natural history and clinical consequences N Engl J Med 2004 10 1118 1129 10.1056/NEJMra031087 15014185

[B3] Liaw YF Chu CM Hepatitis B virus infection Lancet 2009 10 582 592 10.1016/S0140-6736(09)60207-5 19217993

[B4] Kao JH Molecular epidemiology of hepatitis B virus Korean J Intern Med 2011 10 255 261 10.3904/kjim.2011.26.3.255 22016585 PMC3192197

[B5] Guidotti LG Matzke B Schaller H Chisari FV High level hepatitis B virus replication in transgenic mice J Virol 1995 10 6158 6169 7666518 10.1128/jvi.69.10.6158-6169.1995 PMC189513

[B6] Araki K Miyazaki J Hino O Tomita N Chisaka O Expression and replication of hepatitis B virus genome in transgenic mice Proc Natl Acad Sci USA 1989 10 207 211 2911569 10.1073/pnas.86.1.207 PMC286433

[B7] Moriyama T Guilhot S Klopchin K Moss B Pinkert CA Immunobiology and pathogenesis of hepatocellular injury in hepatitis B virus transgenic mice Science 1990 10 361 364 10.1126/science.1691527 1691527

[B8] Larkin J Clayton M Sun B Perchonock CE Morgan JL Hepatitis B virus transgenic mouse model of chronic liver disease Nat Med 1999 10 907 912 10.1038/11347 10426314

[B9] Böcher WO Marcus H Shakarchy R Dekel B Shouval D Antigen-specific B and T cells in human/mouse radiation chimera following immunization in vivo Immunology 1999 10 634 641 10.1046/j.1365-2567.1999.00704.x 10233752 PMC2326778

[B10] Ilan E Burakova T Dagan S Nussbaum O Lubin I The hepatitis B virus-trimera mouse: a model for human HBV infection and evaluation of anti-HBV therapeutic agents Hepatology 1999 10 553 562 10.1002/hep.510290228 9918935

[B11] Dandri M Burda MR Török E Pollok JM Iwanska A Repopulation of the mouse liver with human hepatocytes and in vivo infection with hepatitis B virus Hepatology 2001 10 981 988 10.1053/jhep.2001.23314 11283864

[B12] Huang LR Wu HL Chen PJ Chen DS An immunocompetent mouse model for the tolerance of human chronic hepatitis B virus infection Proc Natl Acad Sci USA 2006 10 17862 17867 10.1073/pnas.0608578103 17095599 PMC1635544

[B13] Takehara T Suzuki T Ohkawa K Hosui A Jinushi M Viral covalently closed circular DNA in a non-transgenic mouse model for chronic hepatitis B virus replication J Hepat 2006 10 267 274 10.1016/j.jhep.2005.07.030 16226822

[B14] Cobleigh MA Buonocore L Uprichard SL Rose JK Robek MD A vesicular stomatitis virus-based hepatitis B virus vaccine vector provides protection against challenge in a single dose J Virol 2010 10 7513 7522 10.1128/JVI.00200-10 20504927 PMC2897621

[B15] Lin YJ Huang LR Yang HC Tzeng HT Hsu PN Hepatitis B virus core antigen determines viral persistence in a C57BL/6 mouse model Proc Natl Acad Sci USA 2010 10 9340 9345 10.1073/pnas.1004762107 20439715 PMC2889105

[B16] Yang PL Althage A Chung J Maier H Wieland S Immune effectors required for hepatitis B virus clearance Proc Natl Acad Sci USA 2010 10 798 802 10.1073/pnas.0913498107 20080755 PMC2818933

[B17] Yin Y Wu C Song J Wang J Zhang E DNA Immunization with Fusion of CTLA-4 to Hepatitis B Virus (HBV) Core Protein Enhanced Th2 Type Responsesand Cleared HBV with an Accelerated Kinetic PLoS One 2011 10 1 11 10.1371/journal.pone.0022524 PMC3142188 21799884

[B18] Wu C Deng W Deng L Cao L Qin B Amino acid substitutions at the positions 122 and 145 of hepatitis B surface antigen (HBsAg) determine the antigenicity and immunogenicity of HBsAg and influence in vivo HBsAg clearance J Virol 2012 10 4658 4669 10.1128/JVI.06353-11 22301154 PMC3318601

[B19] Tillmann HL Antiviral therapy and resistance with hepatitis B virus World J Gastroenterol 2007 10 125 140 17206760 10.3748/wjg.v13.i1.125 PMC4065869

[B20] Zoulim F Infection Hepatitis B virus resistance to antiviral drugs: where are we going? Liver Int 2011 10 111 116 21205147 10.1111/j.1478-3231.2010.02399.x PMC3096621

[B21] Haasnoot PC Cupac D Berkhout B Inhibition of virus replication by RNA interference J Biomed Sci 2003 10 607 616 14576463 10.1159/000073526

[B22] Randall G Rice CM Interfering with hepatitis C virus RNA replication Virus Res 2004 10 19 25 10.1016/j.virusres.2004.01.011 15068876

[B23] Wu J Nandamuri KM Inhibition of hepatitis viral replication by siRNA Expert Opin Biol Ther 2004 10 1649 1659 10.1517/14712598.4.10.1649 15461576

[B24] Li BJ Tang Q Cheng D Qin C Xie FY Using siRNA in prophylactic and therapeutic regimens against SARS coronavirus in Rhesus macaque Nat Med 2005 10 944 951 16116432 10.1038/nm1280 PMC7095788

[B25] Tang H Oishi N Kaneko S Murakami S Molecular function and biological roles of hepatitis B virus x protein Cancer Sci 2006 10 977 983 10.1111/j.1349-7006.2006.00299.x 16984372 PMC11159107

[B26] Wei Y Neuveut C Tiollais P Buendia MA Molecular biology of the hepatitis B virus and role of the X gene Pathol Biol 2010 10 267 272 10.1016/j.patbio.2010.03.005 20483545

[B27] Bouchard MJ Schneider RJ The enigmatic X gene of hepatitis B virus J Virol 2004 10 12725 12734 10.1128/JVI.78.23.12725-12734.2004 15542625 PMC524990

[B28] Meng Z Qiu S Zhang X Wu J Schreiter T Inhibition of woodchuck hepatitis virus gene expression in primary hepatocytes by siRNA enhances the cellular gene expression Virology 2009 10 88 96 10.1016/j.virol.2008.11.012 19064272

[B29] Tseng TC Kao JH HBV genotype and clinical outcome of chronic hepatitis B facts and puzzles Gastroenterology 2008 10 1272 1273 10.1053/j.gastro.2007.12.046 18395116

[B30] Kao JH Chen PJ Lai MY Chen DS Hepatitis B genotypes correlate with clinical outcomes in patients with chronic hepatitis B Gastroenterology 2000 10 554 559 10.1016/S0016-5085(00)70261-7 10702206

[B31] Yuan J Zhou B Tanaka Y Kurbanov F Orito E Hepatitis B virus (HBV) genotypes/subgenotypes in china: mutations in core promoter and precore/core and their clinical implications J Clin Virol 2007 10 87 93 10.1016/j.jcv.2007.03.005 17451999

[B32] Durantel D Carrouée-Durantel S Werle-Lapostolle B Brunelle MN Pichoud C A new strategy for studying in vitro the drug susceptibility of clinical isolates of human hepatitis B virus Hepatology 2004 10 855 864 15382118 10.1002/hep.20388

[B33] Yang H Westland C Xiong S Delaney WE In vitro antiviral susceptibility of full-length clinical hepatitis B virus isolates cloned with a novel expression vector Antiviral Res 2004 10 27 36 10.1016/j.antiviral.2003.07.003 14670591

[B34] Lu M Isogawa M Xu Y Hilken G Immunization with the gene expressing woodchuck hepatitis virus nucleocapsid protein fused to cytotoxic-T-lymphocyte-associated antigen 4 leads to enhanced specific immune responses in mice and woodchucks J Virol 2005 10 6368 6376 10.1128/JVI.79.10.6368-6376.2005 15858020 PMC1091665

[B35] Kuhns MC Kleinman SH McNamara AL Rawal B Glynn S Lack of correlation between HBsAg and HBV DNA levels in blood donors who test positive for HBsAg and anti-HBc: implications for future HBV screening policy Transfusion 2004 10 1332 1339 10.1111/j.1537-2995.2004.04055.x 15318857

[B36] Manesis EK Hadziyannis E Angelopoulou OP Hadziyannis SJ Prediction of treatment-related HBsAg loss in HBeAg-negative chronic hepatitis B: a clue from serum HBsAg levels Antivir Ther 2007 10 73 82 17503750

[B37] Wiegand J Wedemeyer H Finger A Heidrich B Rosenau J A decline in hepatitis B virus surface antigen (hbsag) predicts clearance, but does not correlate with quantitative hbeag or HBV DNA levels Antivir Ther 2008 10 547 554 18672533

[B38] Simonetti J Bulkow L McMahon BJ Homan C Snowball M Clearance of hepatitis B surface antigen and risk of hepatocellular carcinoma in acohort chronically infected with hepatitis B virus Hepatology 2010 10 1531 1537 10.1002/hep.23464 20087968

[B39] Chen CC Ko TM Ma HI Wu HL Xiao X Long-term inhibition of hepatitis B virus in transgenic mice by double-stranded adeno-associated virus 8-delivered short hairpin RNA Gene Ther 2007 10 11 19 10.1038/sj.gt.3302846 16929350

[B40] Yang Z Zhang J Cong H A retrovirus-based system to stably silence GDF-8 expression and enhance myogenic differentiation in human rhabdomyosarcoma cells J Gene Med 2008 10 825 833 10.1002/jgm.1216 18563849

[B41] Sebestyen MG Budker VG Budker T Subbotin VM Zhang G Monahan SD Mechanism of plasmid delivery by hydrodynamic tail vein injection. I. Hepatocyte uptake of various molecules J Gene Med 2006 10 852 873 10.1002/jgm.921 16724360

[B42] Crespo A Peydro A Dasi F Benet M Calvete JJ Revert F Hydrodynamic liver gene transfer mechanism involves transient sinusoidal blood stasis and massive hepatocyte endocytic vesicles Gene Ther 2005 10 927 935 10.1038/sj.gt.3302469 15729372

[B43] Liu F Lei J Vollmer R Huang L M**echanism of liver gene transfer by mechanical massage** Mol Ther 2004 10 452 457 10.1016/j.ymthe.2003.12.003 15006613

[B44] Kobayashi N Nishikawa M Hirata K Takakura Y Hydrodynamics-based procedure involves transient hyperpermeability in the hepatic cellular membrane: implication of a nonspecific process in efficient intracellular gene delivery J Gene Med 2004 10 584 592 10.1002/jgm.541 15133769

[B45] Zamore PD Tuschl T Sharp PA RNAi: double-stranded RNA directs the ATP-dependent cleavage of mRNA at 21 to 23 nucleotide intervals Cell 2000 10 25 33 10.1016/S0092-8674(00)80620-0 10778853

[B46] Baer M Nilsen TW Costigan C Altman S Structure and transcription of a human gene for H1 RNA, the RNA component of human RNase P Nucleic Acids Res 1990 10 97 103 10.1093/nar/18.1.97 2308839 PMC330208

[B47] Brummelkamp TR Bernards R Agami R A System for Stable Expression of Short Interfering RNAs in Mammalian Cells Science 2002 10 550 553 10.1126/science.1068999 11910072

[B48] Cougot D Neuveut C Buendia MA HBV induced carcinogenesis J Clin Virol 2005 10 suppl 1 S75 S78 16461228 10.1016/s1386-6532(05)80014-9

[B49] Hung L Kumar V Specific inhibition of gene expression and transactivation functions of hepatitis B virus X protein and c-myc by small interfering RNAs FEBS Lett 2004 10 210 214 10.1016/S0014-5793(04)00113-9 14988024

[B50] Han Q Zhang C Zhang J Tian Z Reversal of hepatitis B virus-induced immune tolerance by an immunostimulatory 3p-HBx-siRNAs in a retinoic acid inducible gene I-dependent manner Hepatology 2011 10 1179 1189 10.1002/hep.24505 21721030

[B51] Reynolds A Leake D Boese Q Scaringe S Marshall WS Rational siRNA design for RNA interference Nat Biotechnol 2004 10 326 330 10.1038/nbt936 14758366

[B52] Morrissey DV Blanchard K Shaw L Jensen K Lockridge JA Activity of stabilized short interfering RNA in a mouse model of hepatitis B virus replication Hepatology 2005 10 1349 1356 10.1002/hep.20702 15880588

[B53] Morrissey DV Lockridge JA Shaw L Blanchard K Jensen K Potent and persistent in vivo anti-HBV activity of chemically modified siRNAs Nat Biotechnol 2005 10 1002 1007 10.1038/nbt1122 16041363

[B54] Xuan B Qian Z Hong J Huang W EsiRNAs inhibit hepatitis B virus replication in mice model more efficiently than synthesized siRNAs Virus Res 2006 10 150 155 10.1016/j.virusres.2005.12.005 16423421

[B55] Aigner A Gene silencing through RNA interference (RNAi) in vivo: strategies based on the direct application of siRNAs J Biotechnol 2006 10 12 25 10.1016/j.jbiotec.2005.12.003 16413079

[B56] Kim SI Shin D Choi TH Lee JC Cheon GJ Systemic and specific delivery of small interfering RNAs to the liver mediated by apolipoprotein A-I Mol Ther 2007 10 1145 1152 17440441 10.1038/sj.mt.6300168

[B57] Garcia T Li J Sureau C Ito K Qin Y Wands J Tong S Drastic reduction in the production of subviral particles does not impair hepatitis B virus virion secretion J Virol 2009 10 11152 11165 10.1128/JVI.00905-09 19706705 PMC2772768

[B58] Kuhns MC Kleinman SH McNamara AL Rawal B Glynn S Busch MP REDS Study Group Lack of correlation between HBsAg and HBV DNA levels in blood donors who test positive for HBsAg and anti-HBc: implications for future HBV screening policy Transfusion 2004 10 1332 1339 10.1111/j.1537-2995.2004.04055.x 15318857

[B59] Tian YJ Xu Y Zhang ZH Meng ZJ Tan L A substitution at the amino acid position 122 of hepatitis B surface antigen (HBsAg) strongly impairs the antigenicity of HBsAg J Clin Microbiol 2007 10 2971 2978 10.1128/JCM.00508-07 17609325 PMC2045265

[B60] Mendy ME Kaye S van der Sande M Rayco-Solon P Waight PA Application of real-time PCR to quantify hepatitis B virus DNA in chronic carriers in The Gambia Virology 2006 10 23 10.1186/1743-422X-3-23 PMC1482686 16594999

[B61] Lu M Yao X Xu Y Lorenz H Dahmen U Combination of an antiviral drug and immunomodulation against hepadnaviral infection in the woodchuck model J Virol 2008 10 2598 2603 10.1128/JVI.01613-07 18160442 PMC2258919

[B62] Meng Z Xu Y Wu J Tian Y Kemper T Inhibition of hepatitis B virus gene expression and replication by endoribonuclease-prepared siRNA J Virol Methods 2008 10 27 33 10.1016/j.jviromet.2008.02.008 18378325 PMC7112819

[B63] von Freyend MJ Untergasser A Arzberger S Oberwinkler H Drebber U Sequential control of hepatitis B virus in a mouse model of acute, self-resolving hepatitis B J Viral Hepat 2011 10 216 226 10.1111/j.1365-2893.2010.01302.x 20367794

[B64] Zhang X Zhang E Ma Z Pei R Jiang M Modulation of hepatitis B virus replication and hepatocyte differentiation by MicroRNA-1 Hepatology 2011 10 1476 1485 10.1002/hep.24195 21520166

