# Supplementary material for: Inhibition of hepatitis B virus (HBV) gene expression and replication by HBx gene silencing in a hydrodynamic injection mouse model with a new clone of HBV genotype B
Source: Virol J. 2013 Jun 28;10:214. doi: 10.1186/1743-422X-10-214 (PMC3751867; doi:10.1186/1743-422X-10-214)
Supplement: Additional file 1: Figure S1 — Construction procedure for the pBS-HBV1.1B, pBS-HBV1.2B, pBS-HBV1.3B and pAAV-HBV1.3B vectors. (A) The fragments containing 1.1, 1.2 and 1.3 fold over length HBV genomes were amplified from the plasmid pUC-HBV containing a full length genotype B HBV genome (1820-3215-1824 nt, GenBank accession number AY220698.1) and inserted into the PstI and SacI sites of the plasmid pBluescript II KS (+). (B) Schematic representation of the 1.1, 1.2 and 1.3 fold HBV genome used in this study are shown. HBV ORFs as well as enhancers are indicated in different patters. (C) The HBV 1.3 fold over length genome DNA was amplified from pGEM-HBV1.3B and sub-cloned into the NotI site of the pAAV-MCS vector. [file 1743-422X-10-214-S1.ppt]

## Slide 1
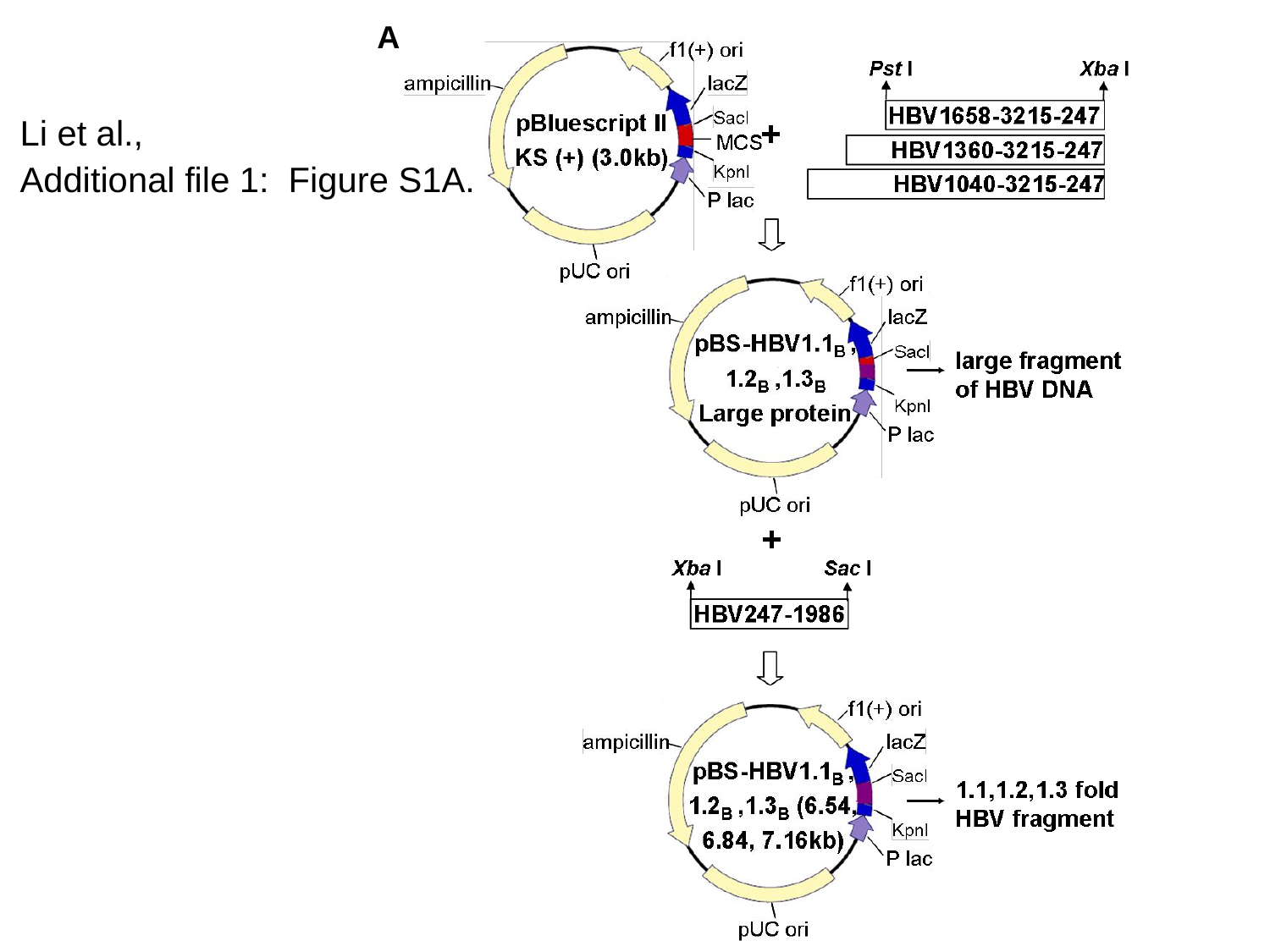

A
Li et al.,
Additional file 1: Figure S1A.

## Slide 2
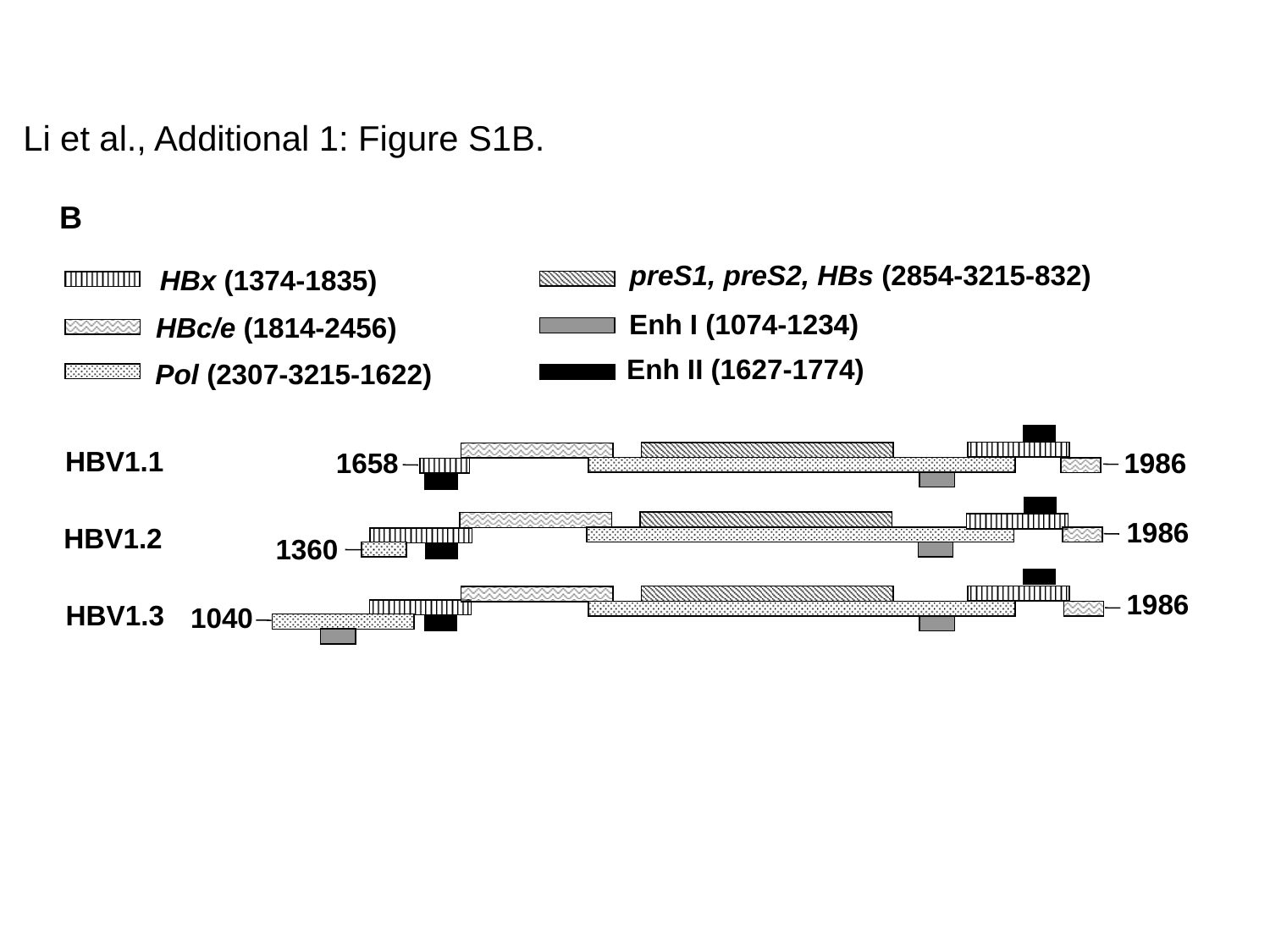

Li et al., Additional 1: Figure S1B.
B
preS1, preS2, HBs (2854-3215-832)
 HBx (1374-1835)
Enh I (1074-1234)
HBc/e (1814-2456)
Enh II (1627-1774)
Pol (2307-3215-1622)
HBV1.1
1658
1986
1986
HBV1.2
1360
1986
HBV1.3
1040

## Slide 3
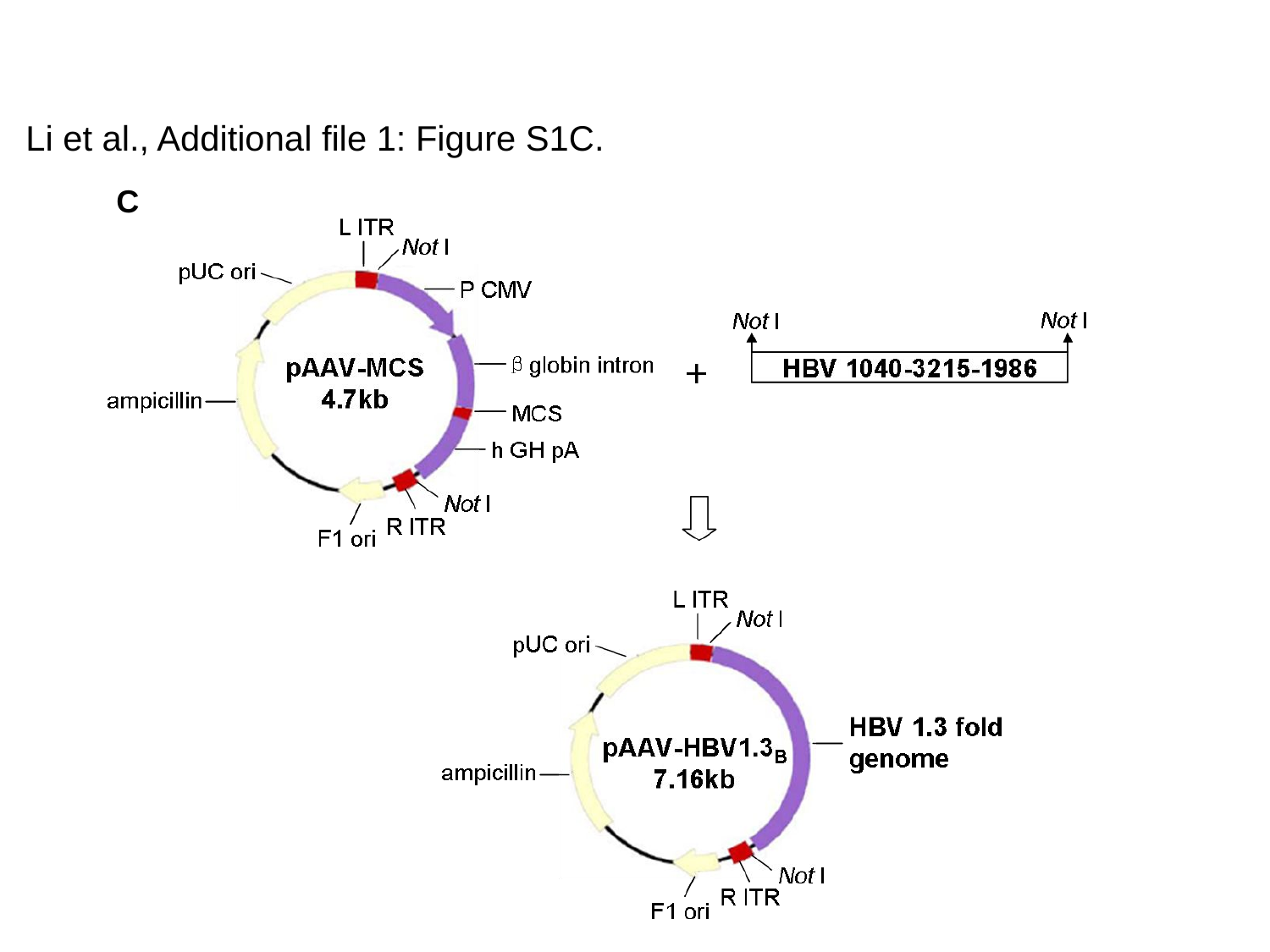

Li et al., Additional file 1: Figure S1C.
C
