# Supplementary figures and images for: Inhibition of hepatitis B virus (HBV) gene expression and replication by HBx gene silencing in a hydrodynamic injection mouse model with a new clone of HBV genotype B
Source: Virol J. 2013 Jun 28;10:214. doi: 10.1186/1743-422X-10-214 (PMC3751867; doi:10.1186/1743-422X-10-214)

## Slide 1
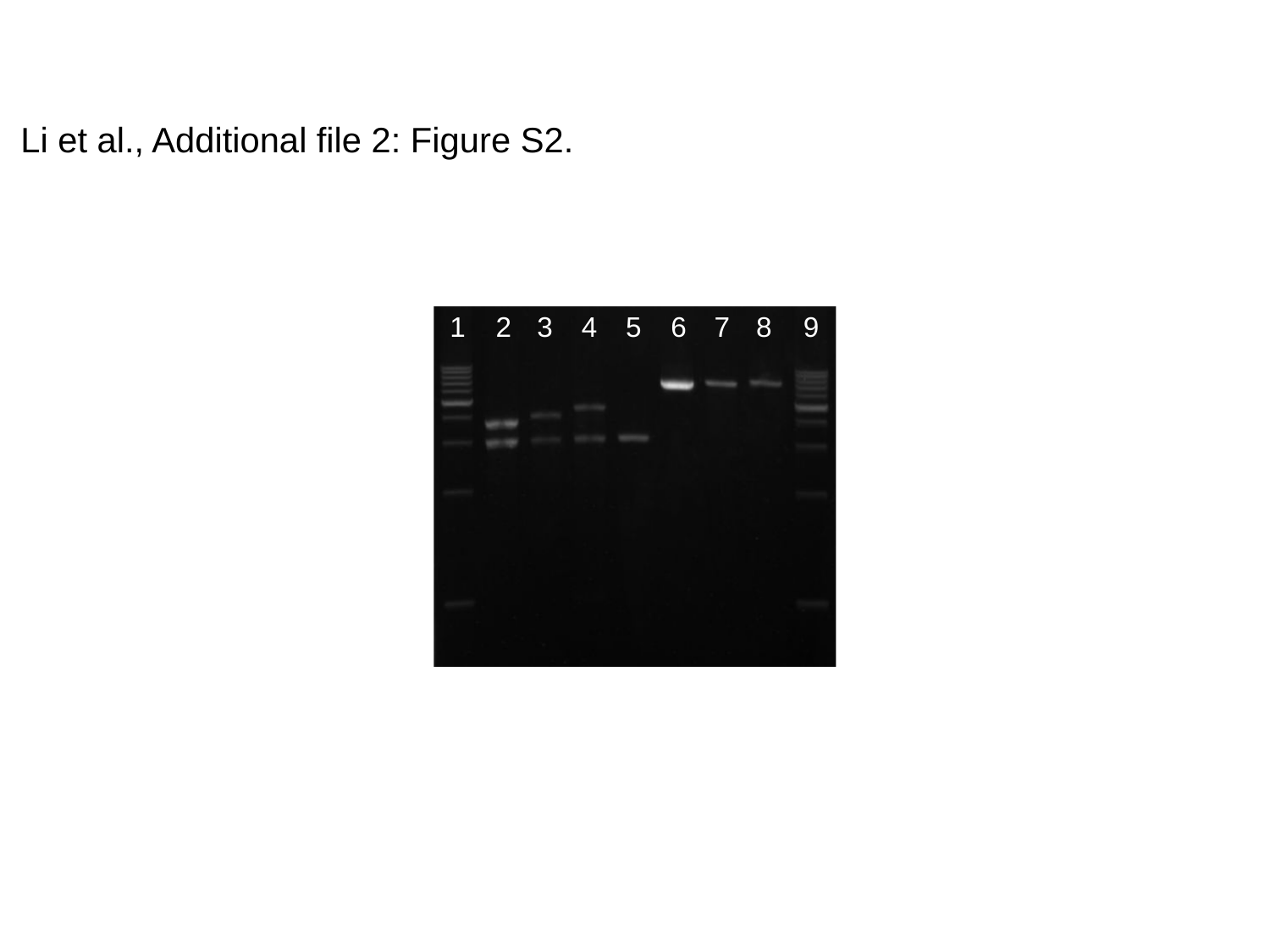

Li et al., Additional file 2: Figure S2.
1
2
3
4
5
6
7
8
9

Supplement: Additional file 2: Figure S2 — Identification of pBS-HBV1.1B, pBS-HBV1.2B and pBS-HBV1.3B. pBS-HBV1.1B, pBS-HBV1.2B and pBS-HBV1.3B were identified by restriction enzyme analysis. Lines 1/9: 1 kb DNA ladder; Lines 2–4: pBS-HBV1.1B, pBS-HBV1.2B and pBS-HBV1.3B were digested with PstI and SacI respectively; Lines 5: pBluescriptII KS (+) was digested with PstI and SacI; Lines 6–8: pBS-HBV1.1B, pBS-HBV1.2B and pBS-HBV1.3B were digested with PstI. [file 1743-422X-10-214-S2.ppt]
